# Distinct influence of parental occupation on cortical thickness and surface area in children and adolescents: Relation to self‐esteem

**DOI:** 10.1002/hbm.25169

**Published:** 2020-10-15

**Authors:** Budhachandra Khundrakpam, Suparna Choudhury, Uku Vainik, Noor Al‐Sharif, Neha Bhutani, Seun Jeon, Ian Gold, Alan Evans

**Affiliations:** ^1^ Montreal Neurological Institute, McGill University Montreal Quebec Canada; ^2^ Ludmer Centre for Neuroinformatics and Mental Health, McGill University Montreal Quebec Canada; ^3^ Division of Social and Transcultural Psychiatry McGill University Montreal Quebec Canada; ^4^ Institute of Psychology, Faculty of Social Sciences University of Tartu Tartu Estonia

**Keywords:** brain development, cortical thickness, parental occupation, self‐esteem, socioeconomic status (SES), surface area

## Abstract

Studies of socioeconomic disparities have largely focused on correlating brain measures with either composite measure of socioeconomic status (SES), or its components—family income or parental education, giving little attention to the component of parental occupation. Emerging evidence suggests that parental occupation may be an important and neglected indicator of childhood and adolescent SES compared to absolute measures of material resources or academic attainment because, while related, it may more precisely capture position in social hierarchy and related health outcomes. On the other hand, although cortical thickness and surface area are brain measures with distinct genetic and developmental origins, large‐scale neuroimaging studies investigating regional differences in interaction of the composite measure of SES or its components with cortical thickness and surface area are missing. We set out to fill this gap, focusing specifically on the role of parental occupation on cortical thickness and surface area by analyzing magnetic resonance imaging scans from 704 healthy individuals (age = 3–21 years). We observed spatially distributed patterns of (parental occupation × age^2^) interaction with cortical thickness (localized at the left caudal middle frontal, the left inferior parietal and the right superior parietal) and surface area (localized at the left orbitofrontal cortex), indicating independent sources of variability. Further, with decreased cortical thickness, children from families with lower parental occupation exhibited lower self‐esteem. Our findings demonstrate distinct influence of parental occupation on cortical thickness and surface area in children and adolescents, potentially reflecting different neurobiological mechanisms by which parental occupation may impact brain development.

## Graphical abstract

Parental occupation may be an important and neglected indicator of childhood and adolescent socioeconomic status (SES) compared to absolute measures of material resources or academic attainment because, while related, it may more precisely capture position in social hierarchy and related health outcomes. By analyzing magnetic resonance imaging scans from 704 healthy individuals (age = 3–21 years), we observed spatially distributed patterns of (parental occupation × age^2^) interaction with cortical thickness (localized at the left caudal middle frontal, the left inferior parietal and the right superior parietal) and surface area (localized at the left orbitofrontal cortex), indicating independent sources of variability. Our findings demonstrate distinct influence of parental occupation on cortical thickness and surface area in children and adolescents, potentially reflecting different neurobiological mechanisms by which parental occupation may impact brain development.

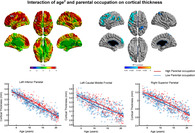

## 1 INTRODUCTION

The experience of living with socioeconomic disadvantage during childhood and adolescence has been consistently linked to pronounced differences in mental and physical health, educational attainment, cognitive, and social–emotional development (Ackerman, Brown, & Izard, 2004; Brooks‐Gunn & Duncan, 1997; Reiss, 2013; van Oort, van der Ende, Wadsworth, Verhulst, & Achenbach, 2011; Vukojević et al., 2017). Poorer families are more likely to experience low birth weight babies, birth defects, fetal alcohol syndrome among other problems, mediated by processes ranging from the experience of racism to poor maternal nutrition and toxic environments in the neighborhood (Aber, Bennett, Conley, & Li, 1997). Children and adolescents from lower socioeconomic conditions are two to three times more likely to develop mental health problems (Reiss, 2013). During childhood, poverty is associated with higher rates of respiratory illnesses, infections (Coultas et al., 1994); while lower cognitive development including academic attainment levels at school (Welsh, Nix, Blair, Bierman, & Nelson, 2010) as well as heightened social–emotional problems have been observed during middle childhood (Brooks‐Gunn & Duncan, 1997), requiring greater mental health care use (Bevaart et al., 2014). Studies have also pointed to greater incidence of mental health problems such as depression among adolescents from poorer families (McLoyd, 1998; Sander & McCarty, 2005; Thapar, Collishaw, Pine, & Thapar, 2012). Genetic studies have started revealing the link between specific genes (GRIN2B) and the worsening of cognitive and behavioral outcomes among children from lower socioeconomic conditions (Riva et al., 2015). Taken together, these data suggest that the developing brain is shaped by the social ecology in which young people live, and as such, have implications for life outcomes.

A major challenge for researchers studying the health impacts of poverty concerns the conceptual and operational definition of poverty, itself, and in particular, childhood poverty (Minujin, Delamonica, Davidziuk, & Gonzalez, 2006). Researchers conducting empirical studies generally use socioeconomic status (SES) as a proxy for poverty. SES is an indicator of a family's access to social and economic resources, and the advantages and social status these resources allow for (Brito & Noble, 2014; Farah, 2017; Vukojević et al., 2017). SES, as it is operationalized in quantitative studies, is a multidimensional construct, most commonly estimated by some permutation of three objective components, which, when concerned with SES of children pertain to the parent(s): income, occupation and education level. Subjective measures of social standing and neighborhood quality are often considered in SES measures as well. As such, SES not only reflects economic resources but also aspects of social hierarchy and prestige.

Parental occupation is one of the three most commonly used proxies for SES, along with family income and parental education. It is important to note that there is great debate around the inconsistency in SES measures. The lack of consistency raises questions about the degree to which these studies (using either a single proxy or composite, multivariate representation of poverty) can be accurately synthesized or compared. The three components of SES are statistically correlated (Farah, 2017), and conceptually related in complex ways (Braveman et al., 2005). For instance, a successful ballet dancer may have low educational attainment (in terms of number of years or higher degrees) but high occupational prestige, or, a professor may have more education and occupational prestige than a mechanic, but lower income. Composite measures of SES such as the frequently used Hollingshead four‐factor index, while commonly used, may obscure distinct processes since the constituent factors (income, occupation, education) correspond to different lived experiences and neural outcomes. Household income level is most commonly used in the literature but defining a child's SES solely from a material standpoint obscures proximal factors that may be better predictive factors of brain impingement such as exposure to environmental toxins or maternal stress, both of which have demonstrated impacts on child cognitive development and yet are not reflected in a definition weighted to purchasing power (D'Angiulli, Lipina, & Olesinska, 2012). Furthermore, income information does not capture the fact that people (especially low‐income groups) may have income in kind, such as food stamps, or crops which are traded. Income can also be an unreliable indicator of social standing for self‐ or transitorily employed workers (McKenzie, 2005). While parents' number of years in education has consistently shown relationships with cognitive outcomes, it may mask quality of education or the resultant occupational prestige as illustrated above. Taken together, parental occupation may be a sensitive indicator of childhood and adolescent SES because it captures position in the social hierarchy, which has consistently been shown to be intimately related to health and life chances (Marmot et al., 1991; Marmot, Rose, Shipley, & Hamilton, 1978; Pinilla, Lopez‐Valcarcel, & Urbanos‐Garrido, 2017). However, we observe that neuroscience studies, thus far, have focused on correlating structural and functional magnetic resonance imaging (MRI) data with either composite measures of SES, family income, or parental education, giving little attention to the component of parental occupation. We therefore set out to build on findings in the social determinants of health literature and examine the relationship between parental occupation and brain structure in children and adolescents.

Given that typical brain development involves intricate processes with regionally specific nonlinear trajectories (Ball, Beare, & Seal, 2019; Giedd et al., 1999; Gogtay et al., 2004; Mills et al., 2016; Shaw et al., 2008; Tamnes et al., 2017) and that SES is a complex, multifactorial phenomenon likely to have differential effects at different time points, it is possible that the brain‐SES relation may vary nonlinearly with age. Indeed, few studies have started reporting such interactions. One such study investigated children and adolescents (age: 5–17 years) and showed *linear* interaction of parental education/family income and age in the left superior temporal gyrus and left inferior frontal gyrus, with a positive relationship between parental education/family income and volume emerging in adolescence (Noble, Houston, Kan, & Sowell, 2012). The same group, using a larger sample of participants (1,148 children and adolescents) from the Pediatric Imaging, Neurocognition and Genetics (PING) study (http://pingstudy.ucsd.edu/Data.php), observed a *curvilinear* association of age and cortical thickness for children from families with lower parental education and family income, while children from families with higher parental education and family income showed *linear* association of age and cortical thickness (Piccolo, Merz, He, Sowell, & Noble, 2016). However, this *nonlinear* interaction of age and parental education/family income was observed for the average cortical thickness (of all brain regions) whereas at region level, this nonlinear interaction was not observed (read as, not significant) except at the left fusiform gyrus which was shown using post hoc analysis.

Previous studies have also shown links between SES, brain development, and cognitive abilities such as language, executive functions (Brito et al., 2017; Noble, Norman, & Farah, 2005). However, to our knowledge, no one has explored the interaction of SES, brain development and self‐esteem. Such a study is important because low self‐esteem in childhood has been shown to be associated with negative health outcomes including depression and anxiety (Orth, Robins, Widaman, & Conger, 2014; Sowislo & Orth, 2013; van Tuijl, de Jong, Sportel, De Hullu, & Nauta, 2014). Self‐esteem may be defined as an individual's subjective evaluation of his/her worth as a person (Donnellan, Trzesniewski, & Robins, 2013). Children from lower SES families may consider themselves as worthless consequently leading to lower levels of self‐care (Poorgholami, Javadpour, Saadatmand, & Jahromi, 2015). In light of these findings, interaction between SES, brain development and self‐esteem is likely possible.

Our study therefore aimed to shed new light on the relationship between SES and brain development, and its relation to cognition in *four* specific ways. First, given that neurodevelopmental trajectories show high regional specificity (Gogtay et al., 2004; Shaw et al., 2008) which in turn relate to cognition (Shaw et al., 2006), it is important to further investigate the nonlinear (SES × age) interaction on brain structure at region level. Second, since brain structure can be fractionated to distinct parameters—cortical thickness and surface area with distinct genetic and developmental origins (Panizzon et al., 2009; Rakic, 1988), it is possible that the interaction of SES and brain development may show distinct regional patterns for cortical thickness and surface area. Third, existing studies that draw on the PING dataset have used family income and parental education as measures of SES. Since different SES measures have differential impacts on brain structure and cognition (Brito et al., 2017; Noble et al., 2015), studying parental occupation will yield further information about the complex interaction between SES and brain development. Fourth, we aimed to extend previous findings of links between SES, brain development, and cognition by using child self‐esteem score as a measure of cognition. We therefore set out to explore *nonlinear* interaction of parental occupation and brain structure (cortical thickness and surface area). Based on previous studies, we hypothesized that parental occupation would show distinct influence on cortical thickness and surface area. Additionally, using scores on child self‐esteem, we set out to test whether the nonlinear interaction of parental occupation and brain structure (cortical thickness and surface area) relate to differential self‐esteem scores. Based on previous findings, we hypothesized that in children from families with lower parental occupation, lower self‐esteem would be associated with decreased cortical thickness.

## 2 MATERIALS AND METHODS

### 2.1 Subjects

The data for the study were obtained from the Pediatric Imaging, Neurocognition and Genetics (PING) study (http://pingstudy.ucsd.edu/Data.php). The PING study (Jernigan et al., 2016) is a comprehensive, publicly shared, data resource for investigating neurocognition, neuroimaging, and genetics in normally developing children and adolescents. The cohort, details described elsewhere (Akshoomoff et al., 2014; Jernigan et al., 2016), comprised of cross‐sectional measurements on 1,493 subjects (aged 3–21 years) aggregated from sites across the United States. Each subject's medical, developmental, behavioral history as well as family medical history and environment were obtained from parental questionnaires.

### 2.2 Socioeconomic factors—parental education, parental occupation, and family income

Socioeconomic factors were recorded as a 7‐point scale rating parental occupation from “unskilled employees” to “higher executives,” 7‐point scale rating parental education from “less than seven years” to “professional degree” and a 12‐point scale rating annual familial income from “less than $5,000” to “over $300,000.” Details of the scales are given in Table 1.

**TABLE 1:** Details of scale used for socioeconomic factors—parental occupation, parental education, and family income

Parental occupation	
Scale	Description
1	Unskilled employees
2	Machine operators and semi‐skilled employees
3	Skilled manual employees
4	Clerical and sales workers, technicians, and owners of little businesses (<2 employees)
5	Administrative personnel, owners of small businesses, and minor professionals
6	Business managers, proprietors of medium‐sized businesses, and lesser professionals
7	Higher executives of large concerns, proprietors, and major professionals

Parental education	
Scale	Description
1	Less than 7 years of school
2	7–9 years of school
3	10–11 years of school
4	High school graduate
5	1–3 years of college (also business school)
6	4‐year college graduate (BA, BS, BM)
7	Professional (MA, MS, ME, MD, PhD, LLD, and the like)

Family income	
Scale	Description
1	<$5,000
2	$5,000–9,999
3	$10,000–19,999
4	$20,000–29,999
5	$30,000–39,999
6	$40,000–49,999
7	$50,000–99,999
8	$100,000–149,999
9	$150,000–199,999
10	$200,000–249,999
11	$250,000–299,999
12	$300,000+

### 2.3 Self‐esteem score

For self‐esteem, the *Rosenberg Self‐Esteem Scale* available with the PING dataset was used. The Rosenberg Self‐Esteem Scale comprises of a 10‐item self‐report measure of global self‐worth by measuring both positive and negative feeling about the self (Rosenberg, 1965). All items are answered using a 4‐point scale format—“strongly agree,” “agree,” “disagree” to “strongly disagree.” Details of the 10‐items are given in Table 2.

**TABLE 2:** Details of 10‐item scale used for Rosenberg Self‐Esteem Scale. Participants are asked to indicate their response to each statement in a four‐point format: (a) strongly agree, (b) agree, (c) disagree, and (d) strongly disagree

Item	Description
1	On the whole, I am satisfied with myself.
2	At times, I think I am no good at all.
3	I feel that I have a number of good qualities.
4	I am able to do things as well as most other people.
5	I feel I do not have much to be proud of.
6	I certainly feel useless at times.
7	I feel that I am a person of worth, at least on an equal plane with others.
8	I wish I could have more respect for myself.
9	All in all, I am inclined to feel that I am a failure.
10	I take a positive attitude toward myself.

### 2.4 Image acquisition and preprocessing

Each site administered a standardized structural MRI protocol. Steps, detailed elsewhere (Jernigan et al., 2016), included a 3D *T*_1_‐weighted inversion prepared RF‐spoiled gradient echo scan using prospective motion correction (PROMO), for cortical and subcortical segmentation; and a 3D *T*_2_‐weighted variable flip angle fast spin echo scan, also using PROMO, for detection and quantification of white matter (WM) lesions and segmentation of CSF.

The CIVET processing pipeline, (http://www.bic.mni.mcgill.ca/ServicesSoftware/CIVET) developed at the Montreal Neurological Institute, was used to compute cortical thickness measurements at 81,924 regions covering the entire cortex. A summary of the steps involved follows; the *T*_1−_weighted image is first nonuniformity corrected, and then linearly registered to the Talairach‐like MNI152 template (established with the ICBM152 dataset). The nonuniformity correction is then repeated using the template mask. The nonlinear registration from the resultant volume to the MNI152 template is then computed, and the transform used to provide priors to segment the image into GM, WM, and cerebrospinal fluid. Inner and outer GM surfaces are then extracted using the constrained Laplacian‐based automated segmentation with proximities algorithm, and cortical thickness is measured in native space using the linked distance between the two surfaces at 81,924 vertices. Each subject's cortical thickness map was blurred using a 30‐mm full‐width at half‐maximum surface‐based diffusion smoothing kernel to impose a normal distribution on the corticometric data, and to increase the signal to noise ratio.

Next, we took several precautions to reduce potential errors and minimize bias during preprocessing of pediatric neuroimaging data. This is because several previous studies have highlighted the necessity for stringent quality control (QC) during preprocessing of pediatric neuroimaging data (Ducharme et al., 2016). This is particularly important because presence of motion artifacts has been shown to be relatively larger in pediatric compared to adult neuroimaging datasets. As such, we followed a stringent QC procedure: QC of the data was performed by two independent reviewers. Only scans with consensus rating from both reviewers were used. As a result of this process, data with motion artifacts, a low signal to noise ratio, artifacts due to hyperintensities from blood vessels, surface–surface intersections, or poor placement of the gray or WM (GM) surface for any reason were excluded. In total, 934 unique participants with MRI scans were obtained from PING in a Box. Out of the total of 934 participants, 29 subjects failed QC procedure. Of these 29 subjects, 13 subjects were excluded before any processing (raw data) due to severe motion and slicing artifacts. The subsequent 16 subjects failed CIVET pipeline (for a number of reasons including presence of bright blood vessels and poor contrast). Thus, 905 participants passed QC procedure. Next, filtering for individuals with information for demographics (age, sex, scanner, parental education, parental occupation, family income, ethnicity) resulted in a final sample of 704 participants. A subset of this sample (*n* = 113) had Rosenberg Self‐Esteem scores. The demographics of the resulting participants used for the study are given in Table 3. Note that, we checked whether there were significant differences in terms of sociodemographic information (a) between children (age, sex, SES measures) in the total PING cohort (*N* = 1,493) and those in the subsample (*N* = 934) and (b) between children excluded due to poor MRI quality (*N* = 29) and those in the final sample (*N* = 704), and between children without (*N* = 591) versus with (*N* = 113) self‐esteem scores. We only observed significant difference (*F* = 18.78, *p* < .001) in age between the total PING data (*N* = 1,493) and the sample with unique MRI data (*N* = 934) such that mean age was greater for the total PING data (*N* = 1,493).

**TABLE 3:** Demographics of the subjects used in the study. Means, with *SD* given in parentheses

Total number of subjects, *N* = 704
Males/females = 364/340
Age = 3–21 (12.4 ± 5) years
Race (GAF)
GAF_Europe = 0–1 (0.64 ± 0.4)
GAF_Africa = 0–1 (0.13 ± 0.2)
GAF_American Indian = 0–0.83 (0.04 ± 0.1)
GAF_East Asian = 0–1 (0.14 ± 0.3)
GAF_Oceania = 0–0.22 (0.005 ± 0.02)
GAF_Central Asia = 0–1 (0.03 ± 0.1)
Number of subjects with Rosenberg Self‐Esteem score, *n* = 113
Rosenberg Self‐Esteem score = 0–2.7 (0.90 ± 0.5)

Abbreviation: GAF, genetic ancestry factor.

Additionally, considering the developing age range (3–21 years), there was a possibility that the adult brain template that we used (MNI152 template established with the ICBM 152 dataset) during MRI preprocessing might not be optimal for the pediatric dataset. We therefore preprocessed the data using an unbiased, age‐appropriate pediatric MRI template established with the NIHPD dataset, age range (4.5–18.5 years) (Fonov et al., 2011), and did the analysis (interaction of age^2^ and parental occupation) on the newly derived cortical thickness values. We obtained very similar results with the reported results, indicating that the use of the adult brain template did not lead to suboptimal preprocessing of the used pediatric dataset. One of the reasons for this might be because the number of very young subjects was relatively lesser.

### 2.5 Statistical analyses

In order to determine interactive effects of SES (as measured with parental occupation) on brain structure (cortical thickness, surface area, and cortical volume) with age, general linear models were constructed for each vertex, respectively, for cortical thickness, surface area, and cortical volume. Models with quadratic age terms were found to fit the data significantly better than models with only lower degree age terms, consistent with earlier findings (Noble et al., 2015; Piccolo et al., 2016). Thus, cortical thickness (/surface area/cortical volume) was modeled as:

$$T_{i}=\text{intercept}+\beta_{1}\mathrm{Age}+\beta_{2}\mathrm{SES}+\beta_{3}\text{Scanner}+\beta_{4}\mathrm{Sex}+\beta_{5}\text{Ethnicity}+\beta_{5}\text{BrainVolume}+\beta_{6}\left(\mathrm{Age}\times \mathrm{SES}\right)+\beta_{7}{\mathrm{Age}}^{2}+\beta_{8}\left({\mathrm{Age}}^{2}\times \mathrm{SES}\right)+\varepsilon_{i}$$

where *i* is a vertex, *ε* is the residual error, and the intercept and the *β* terms are the fixed effects. All statistical analyses were done using the SurfStat toolbox (http://www.math.mcgill.ca/keith/surfstat/). For comparisons, similar analyses were performed for parental education and family income.

At every cortical point, the *t*‐statistic for the association between brain structure (cortical thickness, surface area, and cortical volume) and parental occupation was mapped onto a standard surface; a random field theory (RFT) correction for multiple comparisons (Worsley, Taylor, Tomaiuolo, & Lerch, 2004) was then applied to the resultant map to determine the regions of cortex showing statistically significant association between brain structure and parental occupation. In order to better characterize the age‐related patterns of association between brain structure and parental occupation, as has been done in earlier studies (Brito et al., 2017; Piccolo et al., 2016), we divided the data sample into three groups: Scale 1–3 as low parental occupation group, Scale 4–5 as middle parental occupation group, and Scale 6–7 as high parental occupation group (Table 4a). As can be seen from Table 4b, in our data, subsample with concurrent measures of Self‐Esteem scores, the number of subjects in low parental occupation group for Scale 1–3 was much smaller compared to that of Scale 6–7 (26 compared to 66); so, categorizing Scale 1–5 to 2 groups would lead to disproportionate number of subjects for group comparisons. In view of this, for our study, we categorized Scale 1–5 as the Low parental occupation group. Since there were 9 sites but 12 scanners (one site with two scanners, and another with three scanners), scanner was put as covariate in the analyses. Since parental occupation was categorical, it was dummy coded in the analyses.

**TABLE 4:** Details of groups with low, middle, and high parental occupation. Means, with *SD* given in parentheses

(a) Whole sample				
Group	Subjects (*N*)	Age (years)	Males/females	Parental occupation
Low	120	4.1–21 (11.6 ± 4.9)	63/57	1–3 (1.8 ± 0.9)
Middle	291	3.2–21 (12.2 ± 5.0)	148/143	4–5 (4.6 ± 0.5)
High	293	3.3–21 (12.7 ± 4.9)	153/140	6–7 (6.4 ± 0.5)

(b) Subsample used for studying relation with self‐esteem score				
Group	Subjects	Age (years)	Self‐esteem	Parental occupation
Low	26	4.3–17.3 (10.7 ± 4.1)	0–2.7 (1.1 ± 0.5)	1–3 (1.3 ± 0.7)
Middle	21	4.5–21.0 (11.9 ± 5.1)	0–2.7 (0.9 ± 0.6)	4–5 (4.6 ± 0.5)
High	66	3.3–21.0 (13.0 ± 5.2)	0–2.7 (0.8 ± 0.5)	6–7 (6.4 ± 0.5)

Next, as a post hoc analysis, we explored whether brain structure (cortical thickness, surface area, and cortical volume) at the peak vertices show differential relation with self‐esteem scores for Low and High parental occupation groups. General linear models were used to model brain structure of peak vertex with interaction of parental occupation and self‐esteem scores. Age, sex, scanner, and ethnicity were included as covariates. Similar analyses were performed for parental education and family income.

## 3 RESULTS

### 3.1 (Parental occupation × age^2^) interaction with brain structure (cortical thickness, surface area, and cortical volume)

**FIGURE 1:**
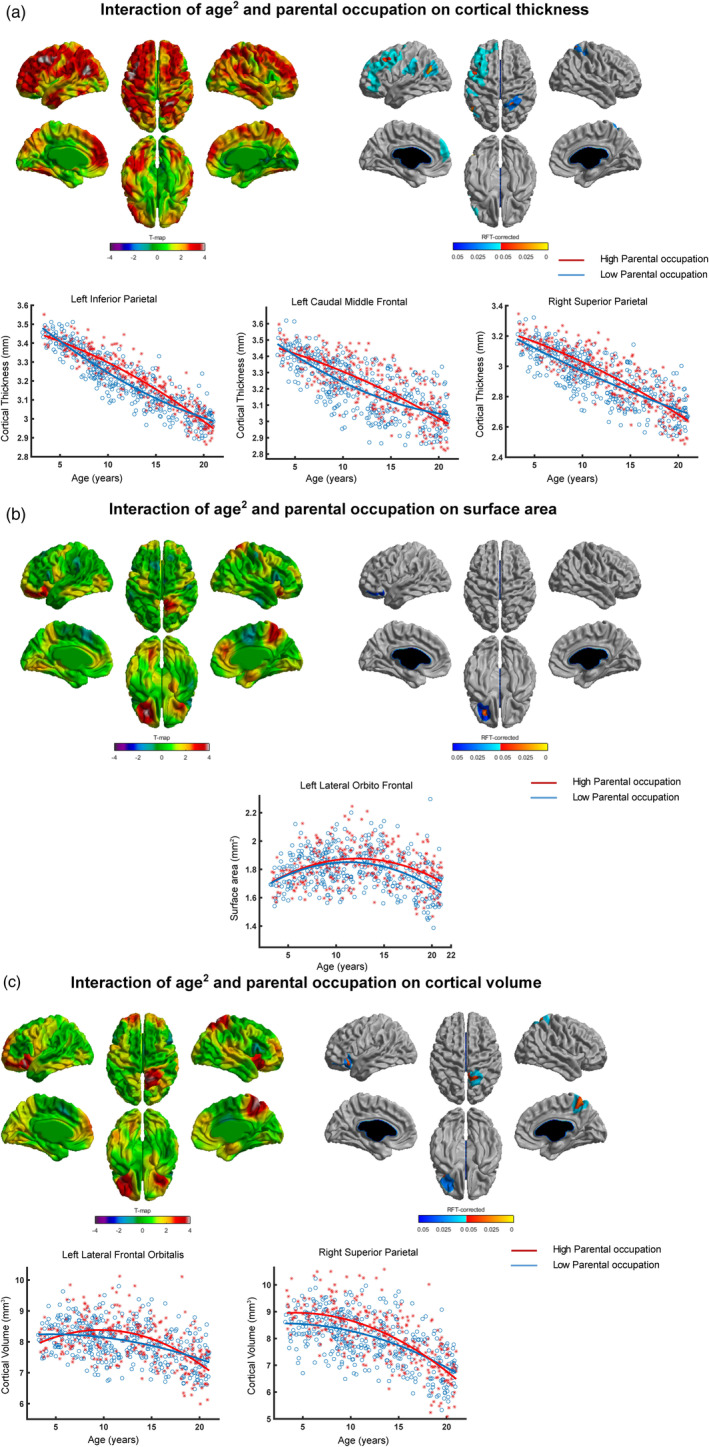
Interaction of (parental occupation × age^2^) and brain structure. Spatially distributed patterns of (parental occupation × age^2^) interaction on (a) cortical thickness, (b) surface area, and (c) cortical volume with the left and right panels showing *t*‐statistics and *p*‐statistics (*p* < .05 after correcting for multiple comparisons using random field theory), respectively. Fitted curves of cortical thickness (a), surface area (b), and cortical volume (c) data are depicted for vertices with maximum *t*‐statistics (see Table 5). Note: Individuals from families with higher parental occupation exhibited greater cortical thickness/surface area/cortical volume for several periods of development when compared to individuals from families with lower parental occupation. Note: x‐Axis = age (years), y‐axis = cortical thickness/surface area/cortical volume

We observed significant (parental occupation × age^2^) interaction (*p* < .05, RFT‐corrected) with cortical thickness in several brain regions localized at the left inferior parietal, the left caudal middle frontal and the right superior parietal cortices (Table 5a, Figure 1a). At the peak vertices, in order to better characterize the (parental occupation × age^2^) interaction with cortical thickness, we plotted cortical thickness for low and high parental occupation groups. Greater cortical thickness was observed for the high parental occupation compared to that of low parental occupation group from 7 till around 16 years.

**TABLE 5:** Peak vertices for significant interaction of age^2^ and parental occupation with (a) cortical thickness, (b) surface area, and (c) cortical volume. The MNI coordinates of the peak vertices with corresponding *t*‐statistics and *p*‐values and brain labels are shown. *X*, *Y*, and *Z* denote MNI coordinates. Left, left hemisphere; right, right hemisphere

(a) With cortical thickness						
*X*	*Y*	*Z*	*T*	*p*	Cluster size	Brain label
−54	−63	22	4.24	.018	580	Left inferior parietal
−37	19	42	4.16	.024	2,399	Left caudal middle frontal
21	−52	67	4.08	.034	391	Right superior parietal

(b) With surface area						
*X*	*Y*	*Z*	*T*	*p*	Cluster size	Brain label
−28	39	−13	3.94	.026	619	Left lateral orbitofrontal

(c) With cortical volume						
*X*	*Y*	*Z*	*T*	*p*	Cluster size	Brain label
−37	25	−10	3.93	.023	839	Left lateral frontal Orbitalis
12	−56	64	3.91	.024	1,013	Right superior parietal

(Parental occupation × age^2^) interaction with surface area was significant only for a cluster in the left orbitofrontal cortex (Table 5b, Figure 1b). There was no group difference in surface area till 14 years, after which high parental occupation group showed greater surface area compared to the low parental occupation group.

For cortical volume, (parental occupation × age^2^) interaction was significant in brain regions localized at the left lateral frontal orbitalis and the right superior parietal cortices (Table 5c, Figure 1c). Greater cortical volume was observed for high parental occupation compared to the low parental occupation group from around 9 to 14 years.

### 3.2 (Parental education × age^2^) interaction with brain structure (cortical thickness, surface area, and cortical volume)

**FIGURE 2:**
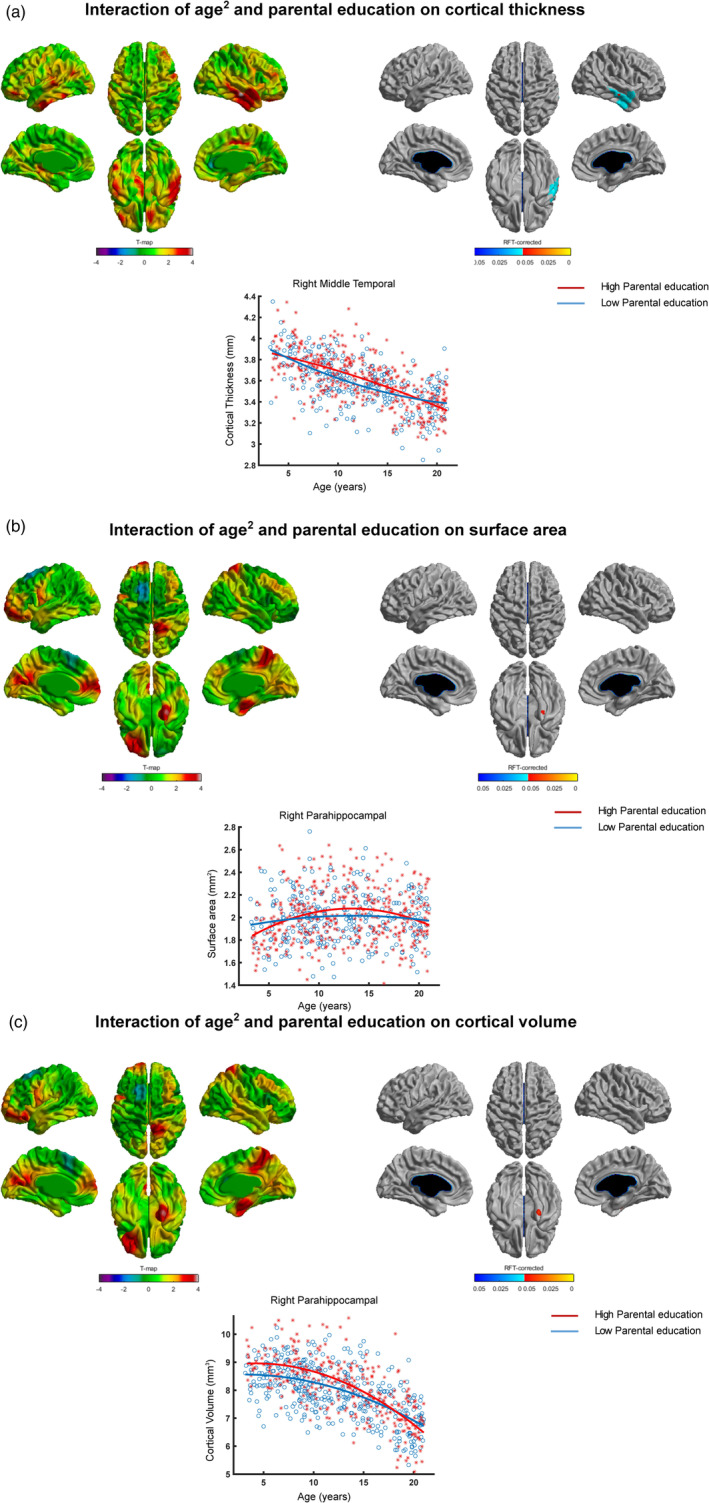
Interaction of (parental education × age^2^) and brain structure. Spatially distributed patterns of (parental education × age^2^) interaction on (a) cortical thickness, (b) surface area, and (c) cortical volume with the left and right panels showing *t*‐statistics and *p*‐statistics (*p* < .05 after correcting for multiple comparisons using random field theory), respectively. Fitted curves of cortical thickness (a), surface area (b), and cortical volume (c) data are depicted for vertices with maximum *t*‐statistics (see Table 6). Note: Individuals from families with higher parental education exhibited greater cortical thickness/surface area/cortical volume for several periods of development when compared to individuals from families with lower parental education. Note: x‐axis = age (years), y‐axis = cortical thickness/surface area/cortical volume

We observed significant (parental education × age^2^) interaction (*p* < .05, RFT‐corrected) with cortical thickness localized in the right middle temporal gyrus (Table 6a, Figure 2a). Greater cortical thickness was observed for the high parental education group compared to that of low parental education group from 7 till around 16 years.

**TABLE 6:** Peak vertices for significant interaction of age^2^ and parental education with (a) cortical thickness, (b) surface area, and (c) cortical volume. The MNI coordinates of the peak vertices with corresponding *t*‐statistics and *p*‐values and brain labels are shown. *X*, *Y*, and *Z* denote MNI coordinates. Left, left hemisphere; right, right hemisphere

(a) With cortical thickness						
*X*	*Y*	*Z*	*T*	*p*	Cluster size	Brain label
67	−16	−15	4.06	.021	920	Right middle temporal

(b) With surface area						
*X*	*Y*	*Z*	*T*	*p*	Cluster size	Brain label
28	−12	−32	3.81	.031	284	Right parahippocampal

(c) With cortical volume						
*X*	*Y*	*Z*	*T*	*p*	Cluster size	Brain label
28	−12	−32	3.80	.033	351	Right parahippocampal

(Parental education × age^2^) interaction with surface area was significant only for a cluster in the right parahippocampal (Table 6b, Figure 2b). Greater surface area was observed for the high parental education group compared to that of low parental education group from 10 till around 16 years.

For cortical volume, (parental education × age^2^) interaction was significant only for a cluster in the right parahippocampal (Table 6c, Figure 2c). Greater cortical volume was observed for high parental education group compared to the low parental education group from 3 to around 14 years.

### 3.3 (Family income × age^2^) interaction with brain structure (cortical thickness, surface area, and cortical volume)

**FIGURE 3:**
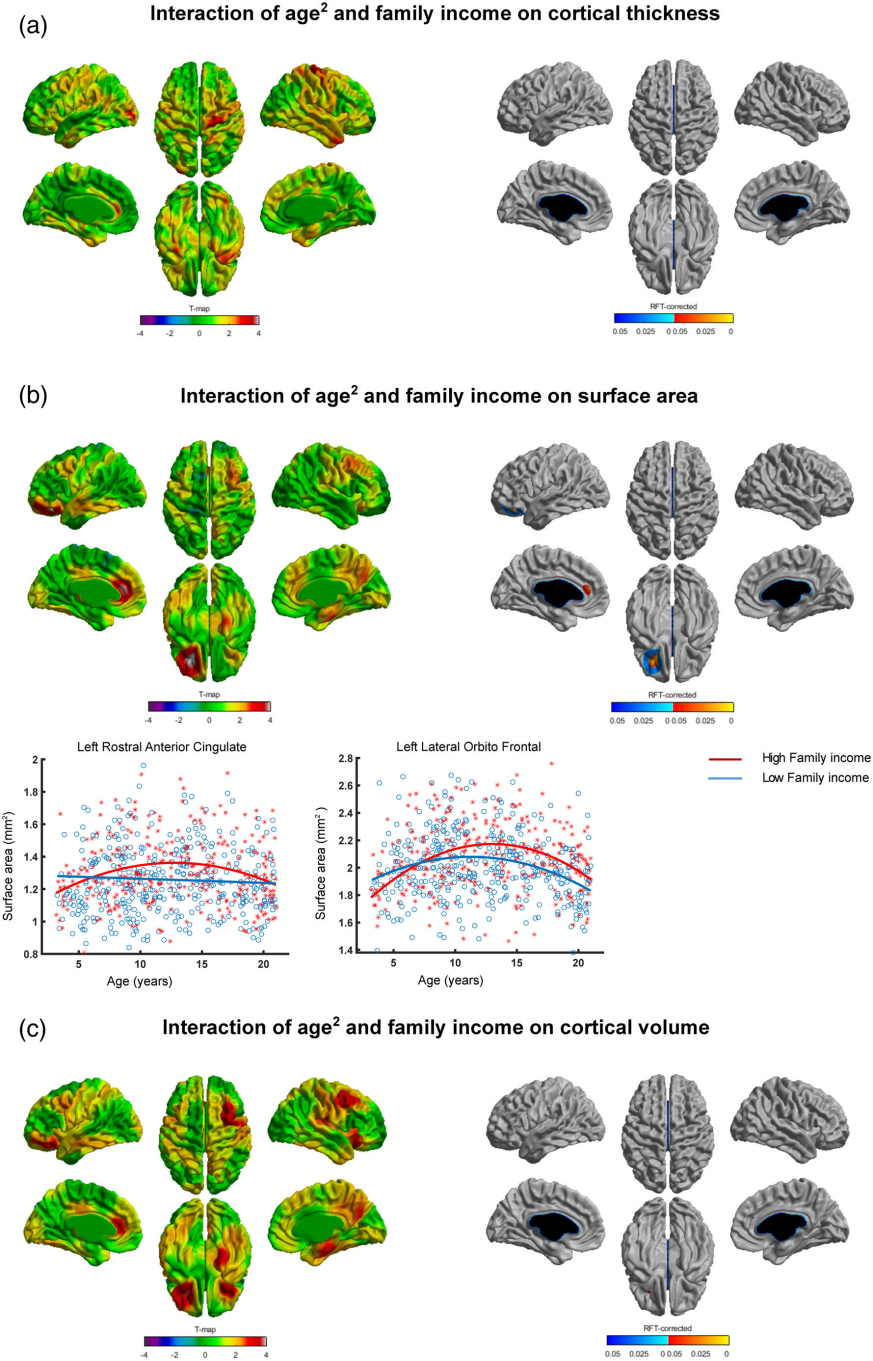
Interaction of (family income × age^2^) and brain structure. Spatially distributed patterns of (family income × age^2^) interaction on (a) cortical thickness, (b) surface area, and (c) cortical volume with the left and right panels showing *t*‐statistics and *p*‐statistics (*p* < .05 after correcting for multiple comparisons using random field theory), respectively. Fitted curves of cortical thickness (a), surface area (b), and cortical volume (c) data are depicted for vertices with maximum *t*‐statistics (see Table 7). Note: Significant interaction of family income and age^2^ was observed for surface area but not for cortical thickness and cortical volume. Individuals from families with higher family income exhibited greater surface area for several periods of development when compared to individuals from families with family income. Note: x‐axis = age (years), y‐axis = surface area

We did not observe any significant (family income × age^2^) interaction with cortical thickness (Figure 3a).

(Family income × age^2^) interaction with surface area was significant at two clusters localized in the left lateral orbitofrontal and left rostral anterior cingulate regions (Table 7, Figure 3b). Greater surface area was observed for the high family income group compared to that of low family income group from around 8 till around 18 years.

**TABLE 7:** Peak vertices for significant interaction of age^2^ and family income with surface area. The MNI coordinates of the peak vertices with corresponding *t*‐statistics and *p*‐values and brain labels are shown. *X*, *Y*, and *Z* denote MNI coordinates. Left, left hemisphere; right, right hemisphere

With surface area						
*X*	*Y*	*Z*	*T*	*p*	Cluster size	Brain label
−25	37	−13	4.12	.013	729	Left lateral orbitofrontal
−4	35	14	3.87	.027	447	Left rostral anterior cingulate

We did not observe any significant (family income × age^2^) interaction with cortical volume (Figure 3c).

### 3.4 Interaction of parental occupation and self‐esteem with brain structure (cortical thickness, surface area, and cortical volume)

**FIGURE 4:**
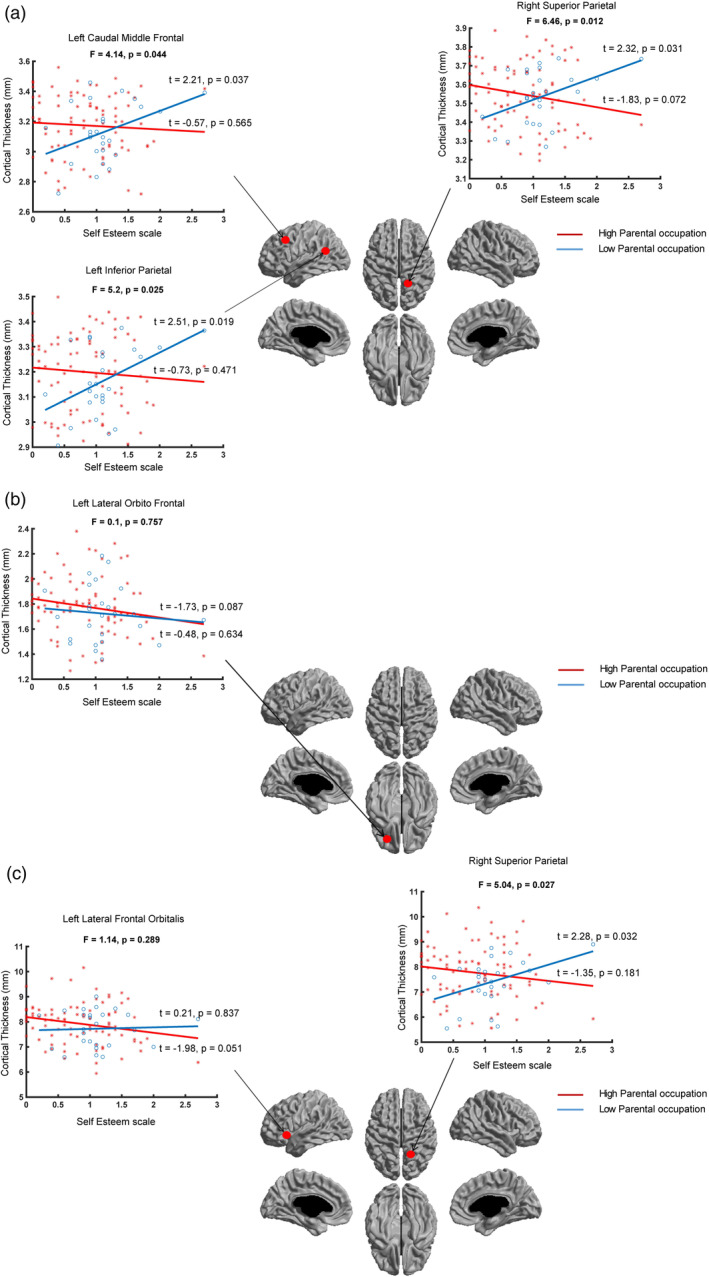
Interaction of parental occupation, self‐esteem, and brain structure. Interaction of parental occupation and self‐esteem for (a) cortical thickness, (b) surface area, and (c) cortical volume at the identified peak vertices (see Table 5) are shown. Significant interaction of parental occupation and self‐esteem was observed with cortical thickness (at all peak vertices) and cortical volume (at one peak vertex), such that individuals from families with lower parental occupation exhibited significant positive association between self‐esteem and cortical thickness and cortical volume. Note: x‐axis = self‐esteem, y‐axis = cortical thickness/surface area/cortical volume

In our post hoc analysis, we studied whether there was interaction of socioeconomic factors (parental occupation/parental education/family income) and self‐esteem with the brain structure (cortical thickness, surface area, and cortical volume) at the identified peak vertices (Tables 5, 6, 7). We observed significant interaction of parental occupation and self‐esteem with cortical thickness at the three peak vertices: the left caudal middle frontal (*F* = 4.14, *p* = .044); the left inferior parietal (*F* = 5.2, *p* = .025); and the right superior parietal (*F* = 6.46, *p* = .012) (Figure 4). For all peak vertices, we observed a similar pattern: (a) significant positive association between self‐esteem and cortical thickness for the low parental occupation group (at the left caudal middle frontal: *t* = 2.21, *p* = .037; at the left inferior parietal: *t* = 2.51, *p* = .019, and at the right superior parietal: *t* = 2.32, *p* = .031) and (b) no significant association between self‐esteem and cortical thickness for the high parental occupation group (at the left caudal middle frontal: *t* = −0.57, *p* = .565; at the left inferior parietal: *t* = −0.73, *p* = .471, and at the right superior parietal: *t* = −1.83, *p* = .072).

There was no significant interaction of parental occupation and self‐esteem with surface area at the peak vertex: the left lateral orbitofrontal (*F* = 0.1, *p* = .757). There was no significant association between child self‐esteem and cortical thickness for the low parental occupation group (*t* = −0.48, *p* = .634) and for the high parental occupation group (*t* = −1.73, *p* = .087).

We observed significant interaction of parental occupation and self‐esteem with cortical volume at one peak vertex: the right superior parietal (*F* = 5.04, *p* = .027), but not at the other peak vertex: the left lateral frontal orbitalis (*F* = 1.14, *p* = .289). At the right superior parietal, there was significant positive association between self‐esteem and cortical volume for the low parental occupation group (*t* = 2.28, *p* = .027), but not for the high parental occupation group (*t* = −1.35, *p* = .181). At the left lateral frontal orbitalis, there was no significant association between self‐esteem and cortical volume for the low parental occupation group (*t* = 0.21, *p* = .837) and the higher SES group (*t* = −1.98, *p* = .051) (Figure 4).

We did not observe any significant interaction of parental education/family income and self‐esteem with brain structure (cortical thickness, surface area, and cortical volume) at the identified peak vertices (Tables 6 and 7).

## 4 DISCUSSION

Using data from a large sample of typically developing children and adolescents (*N* = 704, age = 3–21 years) with a focus on parental occupation, we showed nonlinear (parental occupation × age) interaction with cortical thickness, surface area, and cortical volume. Brain regions with significant (parental occupation × age^2^) interaction with cortical thickness (namely, the left inferior parietal, the left caudal middle frontal and the right superior parietal) were distinct from those with surface area (namely, the left orbitofrontal) consistent with the notion that cortical thickness and surface area have distinct genetic and developmental origins (Panizzon et al., 2009; Rakic, 1988). Upon investigating further, individuals from families with higher parental occupation exhibited greater cortical thickness, surface area, and cortical volume compared to those from families with lower parental occupation during several periods of development. In a subsample with concurrent measures of self‐esteem scores (*n* = 113), cortical thickness of the identified brain regions (namely, the left inferior parietal, the left caudal middle frontal and the right superior parietal) was positively associated with self‐esteem for the low parental occupation group, meaning with lesser cortical thickness, children from low parental occupation group exhibited lower self‐esteem.

Consistent with our data, recent studies have indicated that the impact of socioeconomic factors on the brain changes with age (McDermott et al., 2019; Piccolo et al., 2016; Tooley et al., 2020). In a longitudinal study on infants and toddlers (aged 5 months to 4 years), children from families with lower family income showed slower trajectories of cortical growth compared to that of higher family income during infancy and early childhood (Hanson et al., 2013). Another study reported an interaction of parental education/family income and age such that higher parental education/family income was associated with greater volume in the left superior temporal gyrus and left inferior frontal gyrus of participants during adolescence (Noble et al., 2012). More recently, in another study using the PING dataset, the same group observed nonlinear (curvilinear) cortical trajectories for participants with lower parental education/family income and linear trajectories for participants with higher parental education/family income (Piccolo et al., 2016). However, this *nonlinear* interaction of age and parental education/family income was observed for the average cortical thickness (of all brain regions) whereas at region level, this nonlinear interaction was not significant. Extending these findings, we observed region level (a) quadratic interaction of parental education and age with brain structure (cortical thickness, surface area, and cortical volume, Figure 2a–c), and (b) quadratic interaction of family income and age with surface area (Figure 3b). An addition to the earlier studies is our observation of quadratic (parental occupation × age) interaction with brain structure (cortical thickness, surface area, and cortical volume, Figure 1a–c). Our results indicate greater cortical thickness and cortical volume for high compared to low parental occupation/parental education groups during late childhood and early adolescence (Figures 1a–c and 2a–c). On the other hand, our results suggest greater surface area for high compared to low parental occupation/parental education/family income groups during early and late adolescence (Figures 1b, 2b, and 3b). Taken together, our findings indicate regionally specific nonlinear interactions of age and specific socioeconomic factors (parental occupation/parental education/family income) with brain structure.

Our findings highlight specific as well as common influence of parental occupation, parental education, and family income on brain structure. While cortical thickness and cortical volume showed highly specific brain interaction patterns with specific socioeconomic factors, interaction patterns with surface area displayed largely common brain regions. For cortical thickness, brain regions with significant interaction of age^2^ and parental occupation were widespread (the left caudal middle frontal, left inferior parietal, and right superior parietal, Figure 1a), whereas brain regions with significant interaction of age^2^ and parental education were focal (the right middle temporal, Figure 2a). For cortical volume, brain regions with significant interaction of age^2^ and parental occupation were localized in the left lateral frontal orbitalis and the right superior parietal (Figure 1c), whereas brain regions with significant interaction of age^2^ and parental education were localized in the right parahippocampal (Figure 2c). On the other hand, interaction patterns of socioeconomic factors with surface area displayed largely common brain regions: the left lateral orbitofrontal showed significant interaction of age^2^ and parental occupation (Figure 1b)/family income (Figure 3b), and trend‐level interaction with parental education (Figure 2b). Our findings (with cortical thickness and cortical volume) indicate specific brain regions for specific socioeconomic factors, consistent with previous studies that have shown differential effects of specific socioeconomic factors on brain development (Duncan & Magnuson, 2012; Duncan, Magnuson, & Votruba‐Drzal, 2014).

The highly specific, spatially distributed patterns of (parental occupation × age^2^) interaction for cortical thickness (localized at the left frontal and parietal cortices, and the right parietal cortex, Figure 1a) and surface area (localized at the left orbitofrontal cortex, Figure 1b) exhibited independent sources of variability (read as nonoverlapping), thereby indicating different neurobiological processes. Our findings are consistent with previous studies that have shown distinct genetic and developmental origins for cortical thickness and surface area (Panizzon et al., 2009; Rakic, 1988). In terms of phylogeny, the radial unit hypothesis posits that cortical thickness reflects the number of neurons produced in each ontogenetic column (radial unit) whereas surface area reflects the number of ontogenetic columns (Rakic, 1988). Thus, the spatially distributed nonoverlapping patterns of (parental occupation × age^2^) interaction on cortical thickness and surface area likely reflect the end result of different phylogenetic processes. Finally, since cortical volume is considered the product of cortical thickness and surface area (Raznahan et al., 2011), it is not surprising that we observed patterns of (parental occupation × age^2^) on cortical volume in regions (localized at the left lateral frontal orbitalis and the right superior parietal cortex, Figure 1c) that were identified separately in the case of cortical thickness and surface area.

In terms of understanding the underlying mechanisms behind the distinct trajectories for groups with lower and higher parental occupation, we can leverage the knowledge of neurodevelopmental trajectories that have been useful in understanding typical and atypical brain development (Giedd et al., 1999; Gogtay et al., 2008; Khundrakpam, Lewis, Kostopoulos, Carbonell, & Evans, 2017; Shaw et al., 2006, 2007, 2008; Tamnes et al., 2017; Wendelken et al., 2017; Zielinski et al., 2014). In light of these studies, we can interpret the cortical thickness trajectories for group with lower SES individuals as deviant trajectories with faster thinning during childhood and leveling off in adolescence, consistent with earlier findings (Piccolo et al., 2016). These results align with studies using animal models that have demonstrated mechanisms of early adversity and deviant brain trajectories (Bath, Manzano‐Nieves, & Goodwill, 2016; Fareri & Tottenham, 2016). Specifically, early adversity has been associated with processes such as increased cell death, altered neuronal morphology (Bath et al., 2016; Callaghan & Tottenham, 2016), which in turn may be reflected in the faster cortical thinning during childhood for the group of individuals from families with lower parental occupation.

Consistent with previous studies (Farah, 2017), parental occupation was positively related to parental education (*r* = .63, *p* < .001) and parental income (*r* = .55, *p* < .001). In spite of these relations, we found specific brain regions (with cortical thickness and cortical volume) for specific socioeconomic factors, consistent with previous studies that have shown differential effects of specific socioeconomic factors on brain development (Duncan et al., 2014; Duncan & Magnuson, 2012). More specifically, we observed widespread cortical regions interacting with parental occupation as opposed to more localized cortical regions in case of parental education. Interestingly, interaction of family income with brain structure (cortical thickness, cortical volume) was nonsignificant for any cortical regions. In terms of interpreting our findings, we can build on findings in the social determinants of health literature. For example, our null findings of family income may be because defining a child's SES solely from a material standpoint obscures proximal factors that may be better predictive factors of brain impingement such as exposure to environmental toxins or maternal stress, both of which have demonstrated impacts on child cognitive development (D'Angiulli et al., 2012). Furthermore, income information does not capture the fact that people (especially low‐income groups) may have income in kind, such as food stamps, or crops which are traded. Income can also be an unreliable indicator of social standing for self‐ or transitorily employed workers (McKenzie, 2005). Future studies involving large‐scale neuroimaging data along with separate components of SES are necessary to further elucidate the specificity of neural correlates for distinct components of SES.

Our findings of links between brain structure and self‐esteem of children with parental occupation add to the existing literature of neurocognitive development and socioeconomic disparities (Brito et al., 2017; Noble et al., 2005). Our observations of lower self‐esteem and decreased cortical thickness in children from families with lower parental occupation are in line with the notion that children from families with lower socioeconomic factors may consider themselves as worthless consequently leading to lower levels of self‐care (Poorgholami et al., 2015) and negative health outcomes including depression and anxiety (Orth et al., 2014; Sowislo & Orth, 2013; van Tuijl et al., 2014). The possible reasons for the negative health consequences of lower self‐esteem may be due to peer pressure (children with lower self‐esteem are likely to be influenced by others) as well as deliberating negative aspect of self (Sowislo & Orth, 2013). In light of these studies, it is possible that in children from lower SES families, lower self‐esteem may be associated with decreased cortical thickness. On the other hand, children from families with higher socioeconomic factors may have protective factors (e.g., warmth parent–child interaction) that mitigate the effects of lower self‐esteem, which may be the reason why we did not observe association between cortical thickness and self‐esteem. Although longitudinal studies with large samples are required, nevertheless our findings add a neurobiological link between parental socioeconomic factors and self‐esteem of children.

The main limitation of our study is the use of cross‐sectional data; as such, our findings are correlational rather than causal, and must therefore be interpreted cautiously. It is therefore not clear whether socioeconomic disparities lead to lesser self‐esteem via altered neurodevelopmental trajectories. Nonetheless, the analysis of a large cohort of young people is suggestive of differential effects of socioeconomic factors during development. Further investigations are required to unpack the underlying biological mechanisms and social factors such as family stress, prenatal factors, cognitive deprivation, or toxins. Future studies utilizing longitudinal MRI data from available datasets such as the ABCD (Casey et al., 2018), tracking children over time with social and environmental measures (Zucker et al., 2018), along with animal studies of the possible underlying biological pathways will help better understand the complex interplay of socioeconomic factors, brain and life outcomes (Hackman, Farah, & Meaney, 2010).

In conclusion, our study highlights the need for considering parental occupation as a proxy for social standing. Additionally, our study demonstrates distinct influence of parental occupation on cortical thickness and surface area in children and adolescents, potentially reflecting different neurobiological mechanisms by which parental occupation may impact brain development. Future studies should explore cortical thickness and surface area in isolation to elucidate the neurobiology of socioeconomic factors, particularly in the context of development.

## CONFLICT OF INTEREST

The authors declare no conflict of interest.

## Data Availability

Data used in the study are available from the PING study at NDAR (https://nda.nih.gov/about.html) after providing the required data user agreement.

## References

[hbm25169-bib-0001] Aber, J. L. , Bennett, N. G. , Conley, D. C. , & Li, J. (1997). The effects of poverty on child health and development. Annual Review of Public Health, 18, 463–483. 10.1146/annurev.publhealth.18.1.463 9143727

[hbm25169-bib-0002] Ackerman, B. P. , Brown, E. D. , & Izard, C. E. (2004). The relations between persistent poverty and contextual risk and children's behavior in elementary school. Developmental Psychology, 40, 367–377. 15122963 10.1037/0012-1649.40.3.367

[hbm25169-bib-0003] Akshoomoff, N. , Newman, E. , Thompson, W. K. , McCabe, C. , Bloss, C. S. , Chang, L. , … Jernigan, T. L. (2014). The NIH toolbox cognition battery: Results from a large normative developmental sample (PING). Neuropsychology, 28, 1–10. 24219608 10.1037/neu0000001 PMC3925365

[hbm25169-bib-0004] Ball, G. , Beare, R. , & Seal, M. L. (2019). Charting shared developmental trajectories of cortical thickness and structural connectivity in childhood and adolescence. Human Brain Mapping, 40, 4630–4644. 31313446 10.1002/hbm.24726 PMC6865644

[hbm25169-bib-0005] Bath, K. G. , Manzano‐Nieves, G. , & Goodwill, H. (2016). Early life stress accelerates behavioral and neural maturation of the hippocampus in male mice. Hormones and Behavior, 82, 64–71. 27155103 10.1016/j.yhbeh.2016.04.010 PMC5308418

[hbm25169-bib-0006] Bevaart, F. , Mieloo, C. L. , Wierdsma, A. , Donker, M. C. H. , Jansen, W. , Raat, H. , … Van Oort, F. V. A. (2014). Ethnicity, socioeconomic position and severity of problems as predictors of mental health care use in 5‐ to 8‐year‐old children with problem behaviour. Social Psychiatry and Psychiatric Epidemiology, 49, 733–742. 24077635 10.1007/s00127-013-0761-4

[hbm25169-bib-0007] Braveman, P. A. , Cubbin, C. , Egerter, S. , Chideya, S. , Marchi, K. S. , Metzler, M. , & Posner, S. (2005). Socioeconomic status in health research. Journal of the American Medical Association, 294, 2879–2888. 16352796 10.1001/jama.294.22.2879

[hbm25169-bib-0008] Brito, N. H. , & Noble, K. G. (2014). Socioeconomic status and structural brain development. Frontiers in Neuroscience, 8, 1–12. 25249931 10.3389/fnins.2014.00276 PMC4155174

[hbm25169-bib-0009] Brito, N. H. , Piccolo, L. R. , Noble, K. G. , & Pediatric Imaging, Neurocognition, and Genetics Study . (2017). Associations between cortical thickness and neurocognitive skills during childhood vary by family socioeconomic factors. Brain and Cognition, 116, 54–62. 28377043 10.1016/j.bandc.2017.03.007 PMC6527098

[hbm25169-bib-0010] Brooks‐Gunn, J. , & Duncan, G. J. (1997). The effects of poverty on children. The Future of Children, 7, 55. 9299837

[hbm25169-bib-0011] Callaghan, B. L. , & Tottenham, N. (2016). The stress acceleration hypothesis: Effects of early‐life adversity on emotion circuits and behavior. Current Opinion in Behavioral Sciences, 7, 76–81. 29644262 10.1016/j.cobeha.2015.11.018 PMC5890821

[hbm25169-bib-0012] Casey, B. J. , Cannonier, T. , Conley, M. I. , Cohen, A. O. , Barch, D. M. , Heitzeg, M. M. , … Dale, A. M. (2018). The adolescent brain cognitive development (ABCD) study: Imaging acquisition across 21 sites. Developmental Cognitive Neuroscience, 32, 43–54. 29567376 10.1016/j.dcn.2018.03.001 PMC5999559

[hbm25169-bib-0013] Coultas, D. B. , Gong, H. , Grad, R. , Handler, A. , McCurdy, S. A. , Player, R. , … Westley, M. (1994). Respiratory diseases in minorities of the United States. American Journal of Respiratory and Critical Care Medicine, 149, S93–S131. 8118656 10.1164/ajrccm/149.3_Pt_2.S93

[hbm25169-bib-0014] D'Angiulli, A. , Lipina, S. J. , & Olesinska, A. (2012). Explicit and implicit issues in the developmental cognitive neuroscience of social inequality. Frontiers in Human Neuroscience, 6, 254. 22973216 10.3389/fnhum.2012.00254 PMC3434357

[hbm25169-bib-0015] Donnellan, M. B. , Trzesniewski, K. H. , & Robins, R. W. (2013). Self‐esteem In The Wiley‐Blackwell handbook of individual differences (pp. 718–746). Oxford, England: Wiley‐Blackwell.

[hbm25169-bib-0016] Ducharme, S. , Albaugh, M. D. , Nguyen, T. V. , Hudziak, J. J. , Mateos‐Pérez, J. M. , Labbe, A. , … O'Neill, J. (2016). Trajectories of cortical thickness maturation in normal brain development—The importance of quality control procedures. NeuroImage, 125, 267–279. 26463175 10.1016/j.neuroimage.2015.10.010 PMC4691414

[hbm25169-bib-0017] Duncan, G. J. , & Magnuson, K. (2012). Socioeconomic status and cognitive functioning: Moving from correlation to causation. Wiley Interdisciplinary Reviews: Cognitive Science, 3, 377–386. 26301469 10.1002/wcs.1176

[hbm25169-bib-0018] Duncan, G. J. , Magnuson, K. , & Votruba‐Drzal, E. (2014). Boosting family income to promote child development. The Future of Children, 24, 99–120. 25518705 10.1353/foc.2014.0008

[hbm25169-bib-0019] Farah, M. J. (2017). The neuroscience of socioeconomic status: Correlates, causes, and consequences. Neuron, 96, 56–71. 28957676 10.1016/j.neuron.2017.08.034

[hbm25169-bib-0020] Fareri, D. S. , & Tottenham, N. (2016). Effects of early life stress on amygdala and striatal development. Developmental Cognitive Neuroscience, 19, 233–247. 27174149 10.1016/j.dcn.2016.04.005 PMC4912892

[hbm25169-bib-0021] Fonov, V. , Evans, A. C. , Botteron, K. , Almli, C. R. , McKinstry, R. C. , & Collins, D. L. (2011). Unbiased average age‐appropriate atlases for pediatric studies. NeuroImage, 54, 313–327. 20656036 10.1016/j.neuroimage.2010.07.033 PMC2962759

[hbm25169-bib-0022] Giedd, J. N. , Blumenthal, J. , Jeffries, N. O. , Castellanos, F. X. , Liu, H. , Zijdenbos, A. , … Rapoport, J. L. (1999). Brain development during childhood and adolescence: a longitudinal MRI study. Nature Neuroscience, 2, 861–863. 10491603 10.1038/13158

[hbm25169-bib-0023] Gogtay, N. , Giedd, J. N. , Lusk, L. , Hayashi, K. M. , Greenstein, D. , Vaituzis, A. C. , … Thompson, P. M. (2004). Dynamic mapping of human cortical development during childhood through early adulthood. Proceedings of the National Academy of Sciences of the United States of America, 101, 8174–8179. 15148381 10.1073/pnas.0402680101 PMC419576

[hbm25169-bib-0024] Gogtay, N. , Lu, A. , Leow, A. D. , Klunder, A. D. , Lee, A. D. , Chavez, A. , … Thompson, P. M. (2008). Three‐dimensional brain growth abnormalities in childhood‐onset schizophrenia visualized by using tensor‐based morphometry. Proceedings of the National Academy of Sciences of the United States of America, 105, 15979–15984. 18852461 10.1073/pnas.0806485105 PMC2566993

[hbm25169-bib-0025] Hackman, D. A. , Farah, M. J. , & Meaney, M. J. (2010). Socioeconomic status and the brain: Mechanistic insights from human and animal research. Neuroscience, 11, 651–659. 20725096 10.1038/nrn2897 PMC2950073

[hbm25169-bib-0026] Hanson, J. L. , Hair, N. , Shen, D. G. , Shi, F. , Gilmore, J. H. , Wolfe, B. L. , & Pollak, S. D. (2013). Family poverty affects the rate of human infant brain growth. PLoS One, 8, e80954. 24349025 10.1371/journal.pone.0080954 PMC3859472

[hbm25169-bib-0027] Jernigan, T. L. , Brown, T. T. , Hagler, D. J. , Akshoomoff, N. , Bartsch, H. , Newman, E. , … Pediatric Imaging, Neurocognition and Genetics Study . (2016). The Pediatric Imaging, Neurocognition, and Genetics (PING) data repository. NeuroImage, 124, 1149–1154. 25937488 10.1016/j.neuroimage.2015.04.057 PMC4628902

[hbm25169-bib-0028] Khundrakpam, B. S. , Lewis, J. D. , Kostopoulos, P. , Carbonell, F. , & Evans, A. C. (2017). Cortical thickness abnormalities in autism Spectrum disorders through late childhood, adolescence, and adulthood: A large‐scale MRI study. Cerebral Cortex, 27, 1–11. 28334080 10.1093/cercor/bhx038

[hbm25169-bib-0029] Marmot, M. G. , Rose, G. , Shipley, M. , & Hamilton, P. J. (1978). Employment grade and coronary heart disease in British civil servants. Journal of Epidemiology and Community Health, 32, 244–249. 744814 10.1136/jech.32.4.244 PMC1060958

[hbm25169-bib-0030] Marmot, M. G. , Smith, G. D. , Stansfeld, S. , Patel, C. , North, F. , Head, J. , … Smith, G. D. (1991). Health inequalities among British civil servants: The Whitehall II study. Lancet, 337, 1387–1393. 1674771 10.1016/0140-6736(91)93068-k

[hbm25169-bib-0031] McDermott, C. L. , Seidlitz, J. , Nadig, A. , Liu, S. , Clasen, L. S. , Blumenthal, J. D. , … Raznahan, A. (2019). Longitudinally mapping childhood socioeconomic status associations with cortical and subcortical morphology. The Journal of Neuroscience, 39, 1365–1373. 30587541 10.1523/JNEUROSCI.1808-18.2018 PMC6381251

[hbm25169-bib-0032] McKenzie, D. J. (2005). Measuring inequality with asset indicators. Journal of Population Economics, 18, 229–260.

[hbm25169-bib-0033] McLoyd, V. C. (1998). Socioeconomic disadvantage and child development. The American Psychologist, 53, 185–204. 9491747 10.1037//0003-066x.53.2.185

[hbm25169-bib-0034] Mills, K. L. , Goddings, A.‐L. L. , Herting, M. M. , Meuwese, R. , Blakemore, S.‐J. J. , Crone, E. A. , … Tamnes, C. K. (2016). Structural brain development between childhood and adulthood: Convergence across four longitudinal samples. NeuroImage, 141, 273–281. 27453157 10.1016/j.neuroimage.2016.07.044 PMC5035135

[hbm25169-bib-0035] Minujin, A. , Delamonica, E. , Davidziuk, A. , & Gonzalez, E. D. (2006). The definition of child poverty: A discussion of concepts and measurements. Environment and Urbanization, 18, 481–500.

[hbm25169-bib-0036] Noble, K. G. , Houston, S. M. , Brito, N. H. , Bartsch, H. , Kan, E. , Kuperman, J. M. , … Sowell, E. R. (2015). Family income, parental education and brain structure in children and adolescents. Nature Neuroscience, 18, 773–778. 25821911 10.1038/nn.3983 PMC4414816

[hbm25169-bib-0037] Noble, K. G. , Houston, S. M. , Kan, E. , & Sowell, E. R. (2012). Neural correlates of socioeconomic status in the developing human brain. Developmental Science, 15, 516–527. 22709401 10.1111/j.1467-7687.2012.01147.x PMC6554027

[hbm25169-bib-0038] Noble, K. G. , Norman, M. F. , & Farah, M. J. (2005). Neurocognitive correlates of socioeconomic status in kindergarten children. Developmental Science, 8, 74–87. 15647068 10.1111/j.1467-7687.2005.00394.x

[hbm25169-bib-0039] Orth, U. , Robins, R. W. , Widaman, K. F. , & Conger, R. D. (2014). Is low self‐esteem a risk factor for depression? Findings from a longitudinal study of mexican‐origin youth. Developmental Psychology, 50, 622–633. 23895172 10.1037/a0033817 PMC3815504

[hbm25169-bib-0040] Panizzon, M. S. , Fennema‐Notestine, C. , Eyler, L. T. , Jernigan, T. L. , Prom‐Wormley, E. , Neale, M. , … Kremen, W. S. (2009). Distinct genetic influences on cortical surface area and cortical thickness. Cerebral Cortex, 19, 2728–2735. 19299253 10.1093/cercor/bhp026 PMC2758684

[hbm25169-bib-0041] Piccolo, L. R. , Merz, E. C. , He, X. , Sowell, E. R. , & Noble, K. G. (2016). Age‐related differences in cortical thickness vary by socioeconomic status. PLoS One, 11, 1–18. 10.1371/journal.pone.0162511 PMC5028041 27644039

[hbm25169-bib-0042] Pinilla, J. , Lopez‐Valcarcel, B. G. , & Urbanos‐Garrido, R. M. (2017). Estimating direct effects of parental occupation on Spaniards' health by birth cohort. BMC Public Health, 17, 26. 28056954 10.1186/s12889-016-3997-6 PMC5217274

[hbm25169-bib-0043] Poorgholami, F. , Javadpour, S. , Saadatmand, V. , & Jahromi, M. K. (2015). Effectiveness of self‐care education on the enhancement of the self‐esteem of patients undergoing hemodialysis. Global Journal of Health Science, 8, 132–136. 26383201 10.5539/gjhs.v8n2p132 PMC4804061

[hbm25169-bib-0044] Rakic, P. (1988). Defects of neuronal migration and the pathogenesis of cortical malformations. Progress in Brain Research, 73, 15–37. 3047794 10.1016/s0079-6123(08)60494-x

[hbm25169-bib-0045] Raznahan, A. , Shaw, P. , Lalonde, F. , Stockman, M. , Wallace, G. L. , Greenstein, D. , … Giedd, J. N. (2011). How does your cortex grow? The Journal of Neuroscience, 31, 7174–7177. 21562281 10.1523/JNEUROSCI.0054-11.2011 PMC3157294

[hbm25169-bib-0046] Reiss, F. (2013). Socioeconomic inequalities and mental health problems in children and adolescents: A systematic review. Social Science & Medicine, 90, 24–31. 23746605 10.1016/j.socscimed.2013.04.026

[hbm25169-bib-0047] Riva, V. , Battaglia, M. , Nobile, M. , Cattaneo, F. , Lazazzera, C. , Mascheretti, S. , … Marino, C. (2015). GRIN2B predicts attention problems among disadvantaged children. European Child & Adolescent Psychiatry, 24, 827–836. 25316095 10.1007/s00787-014-0627-7

[hbm25169-bib-0048] Rosenberg, M. (1965). Society and the adolescent self‐image. Princeton, NJ: Princeton University Press.

[hbm25169-bib-0049] Sander, J. B. , & McCarty, C. A. (2005). Youth depression in the family context: Familial risk factors and models of treatment. Clinical Child and Family Psychology Review, 8, 203–219. 16151618 10.1007/s10567-005-6666-3 PMC1352328

[hbm25169-bib-0050] Shaw, P. , Eckstrand, K. , Sharp, W. , Blumenthal, J. , Lerch, J. P. , Greenstein, D. , … Rapoport, J. L. (2007). Attention‐deficit/hyperactivity disorder is characterized by a delay in cortical maturation. Proceedings of the National Academy of Sciences of the United States of America, 104, 19649–19654. 18024590 10.1073/pnas.0707741104 PMC2148343

[hbm25169-bib-0051] Shaw, P. , Greenstein, D. , Lerch, J. , Clasen, L. , Lenroot, R. , Gogtay, N. , … Giedd, J. (2006). Intellectual ability and cortical development in children and adolescents. Nature, 440, 676–679. 16572172 10.1038/nature04513

[hbm25169-bib-0052] Shaw, P. , Kabani, N. J. , Lerch, J. P. , Eckstrand, K. , Lenroot, R. , Gogtay, N. , … Wise, S. P. (2008). Neurodevelopmental trajectories of the human cerebral cortex. The Journal of Neuroscience, 28, 3586–3594. 18385317 10.1523/JNEUROSCI.5309-07.2008 PMC6671079

[hbm25169-bib-0053] Sowislo, J. F. , & Orth, U. (2013). Does low self‐esteem predict depression and anxiety? A meta‐analysis of longitudinal studies. Psychological Bulletin, 139, 213–240. 22730921 10.1037/a0028931

[hbm25169-bib-0054] Tamnes, C. K. , Herting, M. M. , Goddings, A.‐L. , Meuwese, R. , Blakemore, S.‐J. , Dahl, R. E. , … Mills, K. L. (2017). Development of the cerebral cortex across adolescence: A multisample study of inter‐related longitudinal changes in cortical volume, surface area, and thickness. The Journal of Neuroscience, 37, 3402–3412. 28242797 10.1523/JNEUROSCI.3302-16.2017 PMC5373125

[hbm25169-bib-0055] Thapar, A. , Collishaw, S. , Pine, D. S. , & Thapar, A. K. (2012). Depression in adolescence. Lancet, 379, 1056–1067. 22305766 10.1016/S0140-6736(11)60871-4 PMC3488279

[hbm25169-bib-0056] Tooley, U. A. , Mackey, A. P. , Ciric, R. , Ruparel, K. , Moore, T. M. , Gur, R. C. , … Bassett, D. S. (2020). Associations between neighborhood SES and functional brain network development. Cerebral Cortex, 30, 1–19. 31220218 10.1093/cercor/bhz066 PMC7029704

[hbm25169-bib-0057] van Oort, F. V. A. , van der Ende, J. , Wadsworth, M. E. , Verhulst, F. C. , & Achenbach, T. M. (2011). Cross‐national comparison of the link between socioeconomic status and emotional and behavioral problems in youths. Social Psychiatry and Psychiatric Epidemiology, 46, 167–172. 20165830 10.1007/s00127-010-0191-5 PMC3034891

[hbm25169-bib-0058] van Tuijl, L. A. , de Jong, P. J. , Sportel, B. E. , de Hullu, E. , & Nauta, M. H. (2014). Implicit and explicit self‐esteem and their reciprocal relationship with symptoms of depression and social anxiety: A longitudinal study in adolescents. Journal of Behavior Therapy and Experimental Psychiatry, 45, 113–121. 24135033 10.1016/j.jbtep.2013.09.007

[hbm25169-bib-0059] Vukojević, M. , Zovko, A. , Talić, I. , Tanović, M. , Rešić, B. , Vrdoljak, I. , & Splavski, B. (2017). Parental socioeconomic status as a predictor of physical and mental health outcomes in children ‐ literature review. Acta Clinica Croatica, 56, 742–748. 29590731 10.20471/acc.2017.56.04.23

[hbm25169-bib-0060] Welsh, J. A. , Nix, R. L. , Blair, C. , Bierman, K. L. , & Nelson, K. E. (2010). The development of cognitive skills and gains in academic school readiness for children from low‐income families. Journal of Education & Psychology, 102, 43–53. 10.1037/a0016738 PMC2856933 20411025

[hbm25169-bib-0061] Wendelken, C. , Ferrer, E. , Ghetti, S. , Bailey, S. K. , Cutting, L. , & Bunge, S. A. (2017). Frontoparietal structural connectivity in childhood predicts development of functional connectivity and reasoning ability: A large‐scale longitudinal investigation. The Journal of Neuroscience, 37, 8549–8558. 28821657 10.1523/JNEUROSCI.3726-16.2017 PMC5577859

[hbm25169-bib-0062] Worsley, K. J. , Taylor, J. E. , Tomaiuolo, F. , & Lerch, J. (2004). Unified univariate and multivariate random field theory. NeuroImage, 23(Suppl 1), S189–S195. 15501088 10.1016/j.neuroimage.2004.07.026

[hbm25169-bib-0063] Zielinski, B. A. , Prigge, M. B. D. , Nielsen, J. A. , Froehlich, A. L. , Abildskov, T. J. , Anderson, J. S. , … Lainhart, J. E. (2014). Longitudinal changes in cortical thickness in autism and typical development. Brain, 137, 1799–1812. 24755274 10.1093/brain/awu083 PMC4032101

[hbm25169-bib-0064] Zucker, R. A. , Gonzalez, R. , Feldstein Ewing, S. W. , Paulus, M. P. , Arroyo, J. , Fuligni, A. , … Wills, T. (2018). Assessment of culture and environment in the adolescent brain and cognitive development study: Rationale, description of measures, and early data. Developmental Cognitive Neuroscience, 32, 107–120. 29627333 10.1016/j.dcn.2018.03.004 PMC6436615

